# P-1899. Specialist training in infectious diseases in Europe

**DOI:** 10.1093/ofid/ofaf695.2068

**Published:** 2026-01-11

**Authors:** Jon Salmanton-Garcia, António Guerra, Jean Paul, Eoghan de Barra, Søren Jensen-Fangel, Carlo Torti, Christian Kraef, José María Miró, Annelies Verbon, Oliver A Cornely

**Affiliations:** University Hospital Cologne, Cologne, Germany, Cologne, Nordrhein-Westfalen, Germany; Infectious Diseases Department, Centro Hospitalar do Baixo Vouga – Aveiro, Portugal, Aveiro, Aveiro, Portugal; University Grenoble Alpes, Infectious diseases department, Grenoble, France, grenoble, Rhone-Alpes, France; 6Department of Infectious Diseases, Beaumont Hospital, Beaumont, Dublin 9, Ireland7Department of International Health and Tropical Medicine, Royal College of Surgeons in Ireland, Dublin, Ireland, Dublin, Dublin, Ireland; Department of Infectious Diseases, Aarhus University Hospital, Denmark, Copenhagen, Hovedstaden, Denmark; Università Cattolica del Sacro Cuore, Rome, Italy, rome, Lazio, Italy; CHIP, Rigshospitalet, University of Copenhagen, Copenhagen, Denmark, Copenhagen, Hovedstaden, Denmark; Department of Infectious Diseases, Hospital Clínic-Institut d'Investigacions Biomèdiques August Pi I Sunyer, University of Barcelona, Barcelona, Spain, barcelona, Catalonia, Spain; Department of Microbiology and Infectious Diseases, Erasmus MC, University Medical Centre, Rotterdam, Netherlands, utrecht, Utrecht, Netherlands; University of Cologne, Faculty of Medicine and University Hospital Cologne, Cologne, Nordrhein-Westfalen, Germany

## Abstract

**Background:**

Infectious diseases (ID) remain a global health challenge, requiring specialized training and strong systems. While undergraduate medical education in Europe is standardized, postgraduate ID training varies. Since 2018, UEMS-ID has outlined core competencies to harmonize education. This study explores current trends and disparities in European ID training to inform policy and improve preparedness.

**Methods:**

Delegates to the ID Section and Board of the European Union of Medical Specialists (UEMS) entered national data on a web-based survey tool in late 2023-early 2024. Results were compared to UEMS recommendations on the structure and content of postgraduate training in ID in Europe (2018), and to results of a similar survey in early 2021.

**Results:**

Responses were received from all 36 countries; 27 (75%) recognise ID as an independent speciality and 8 (22.2%) as a subspeciality. Spain does not officially recognise the speciality. Paediatric ID was recognised in 18/36 (50%) of countries. The number of adult ID specialists varied from 78.8 per million inhabitants in Sweden to 0.6 in Germany. Compared to 2021, the number of ID trainees had expanded by more than 75% in Finland, Greece, Ireland, Italy and Turkey and fallen by more than 50% in Latvia, the Netherlands and Poland. Only 8/32 (25%) countries provide the minimum recommended 6 months of training in medical microbiology. Assessment methods included log-books/portfolios in 26/32 (81.3%), workplace-based assessments in 16/32 (50%) and final examinations in 26/32 (81.3%).

**Conclusion:**

There has been little change since 2021 in speciality status or in structure and content of training programmes across Europe. There have been large increases in training position numbers in several countries, possibly in response to COVID-19. Continued low compliance with the 2018 recommendations to increase exposure to medical microbiology during training highlights the slow pace of change. Logistic barriers to change and harmonization across Europe remain and are discussed in the context of published concerns of trainees.

**Disclosures:**

Jon Salmanton-Garcia, MSc, MPH, PhD, menarini, gilead, astrazeneca, pfizer: Honoraria Oliver A. Cornely, Prof. Dr., Al-Jazeera Pharmaceuticals/Hikma: Honoraria|Basilea: Advisor/Consultant|Cidara: Advisor/Consultant|Cidara: Board Member|Cidara: Grant/Research Support|Elion: Advisor/Consultant|F2G: Grant/Research Support|Gilead: Advisor/Consultant|Gilead: Grant/Research Support|Gilead: Honoraria|GlaxoSmithKline: Advisor/Consultant|GlaxoSmithKline: Honoraria|Grupo Biotoscana/United Medical/Knight: Honoraria|Melinta: Advisor/Consultant|Melinta: Board Member|MSD: Honoraria|Mundipharma: Advisor/Consultant|Mundipharma: Grant/Research Support|Mundipharma: Honoraria|Pfizer: Advisor/Consultant|Pfizer: Grant/Research Support|Pfizer: Honoraria|Pulmocide: Board Member|Scynexis: Advisor/Consultant|Scynexis: Grant/Research Support|Shionogi: Advisor/Consultant|Shionogi: Honoraria

